# Brain signatures of error awareness during cognitive tasks for humans in the flight environment

**DOI:** 10.3389/fnins.2022.1007258

**Published:** 2022-11-03

**Authors:** Peng Zhang, Juan Yan, Zhongqi Liu, Qianxiang Zhou

**Affiliations:** ^1^School of Biological Science and Medical Engineering, Beihang University, Beijing, China; ^2^Beijing Advanced Innovation Center for Biomedical Engineering, Beihang University, Beijing, China; ^3^China CDC Key Laboratory of Radiological Protection and Nuclear Emergency, Chinese Center for Disease Control and Prevention, National Institute for Radiological Protection, Beijing, China

**Keywords:** error awareness, EEG, event-related potential, error positivity, pilot, flight environment

## Abstract

At present, many scientific experiments are carried out in extreme conditions. Pilots need to perform high-intensity tasks for a long time. Human error is an essential factor affecting mission execution. To deeply study the physiological characteristics of different erroneous states of consciousness, we used an improved double-choice Oddball paradigm to collect brain electrophysiological signals of volunteers and pilots in missions and analyze event-related potential (ERP), time-frequency, and brain function spectrum, extracting EEG indicators sensitive to error awareness. The results showed that, in the 300∼500 ms time window, the error awareness type was correlated with Pe amplitude. Meanwhile, the time-frequency and brain functional spectrum analysis showed that the amplitude of the aware errors α-ERS oscillation, the functional spectral density of the α-band, and the uncertain errors were more prominent than unaware errors. The error awareness of the pilots showed the same EEG sensitivity characteristics in flight as in the ground volunteer experiment, and the characteristic sensitivity value was higher than that of the ground participants. We analyzed the EEG indicators sensitive to error awareness and determined the differences in EEG characteristics when pilots have error awareness on the ground and in flight. This study provides theoretical guidance for the follow-up research on the intervention measures against error awareness and determines the target point positioning.

## Introduction

In recent years, many scientific experiments have been carried out in the flight environment (Romanell et al., 2020). Therefore, pilots must perform various tasks with high cognitive requirements when facing a mental load for a long time in the flight environment (Connaboy et al., 2020). For pilots to complete operational tasks, maintain safety, and ensure mission success, continuous attention, response inhibition, and other cognitive functions are necessary. Human error is an essential factor affecting task execution. According to relevant statistics, more than 60% of all accidents at home and abroad are related to human error, and the resulting disaster and significant accident rate have reached 80%. In aerospace, the proportion of accidents caused by human error is higher due to the high risk and high complexity of the operation. For example, in severe accidents in the aerospace field, human error factors are as high as 80∼85% (NASA, 1994). In aviation flight accidents, the proportion of accidents caused by human error factors accounts for more than 70%. The occurrence of human error is often related to the environment (Bes, 1999; Youn et al., 2019). For example, pilots left the ground to go to the flight environment and were in a highly closed environment with high pressure and workload for a long time (Alperin et al., 2017). Social isolation may lead to neurological deficits, and a variety of stressors may affect human emotions and cognition, leading to human errors in pilots’ performance of operational tasks (Romanell et al., 2020; Wåhlin et al., 2020).

Many scholars have used event-related potential (ERP) to study the potential neurocognitive basis of error awareness. In these studies, errors in simple selection tasks were observed to have a negative peak about 0–100 ms after the response, which has a frontal, central scalp distribution, known as error negativity (Ne) (Falkenstein, 1990) [now more commonly known as error-related negativity (ERN) (Gehring et al., 1993)]. The central parietal region’s subsequent deflection is called a positive error wave (Pe) (Falkenstein et al., 1991). In the study of error awareness based on EEG, the extent to which ERN and Pe are affected by error awareness has been widely explored by researchers. Some studies (Herrmann et al., 2004; Vocat et al., 2008) used LORETA source localization technology to analyze the brain sources corresponding to ERN and Pe. It was found that ERN was significantly activated in the anterior auxiliary motor area and ACC tail; Pe is more from the beak of ACC. In reverse scanning tasks, Klein used functional magnetic resonance imaging to study the brain regions related to error awareness (Klein et al., 2007). The results showed that error awareness was related bilaterally to the rostral cingulate zone (RCZ), the pre-supplementary motor area (pre-SMA), and the insular cortex. Conscious errors were shown to be more active in the left anterior inferior insular cortex than unaware errors. In addition, it was found that the wrong hemodynamics occurred only after the adjustment of the rostral gyrus, which was also related to the wrong hemodynamics. These results suggest that the rostral part of the cingulate gyrus alone is not enough to produce error awareness. Its signal can only help adjust its subsequent speed-accuracy tradeoff when it is aware of the error. The correct response negativity (CRN) is similar to the ERN and has an apparent negative waveform in the correct response test.

Early studies have established a strong relationship between α-power changes and perceptual tasks (Jasper and Shagass, 1941). The oscillatory activity in the α-band (8∼13 Hz) is a large-scale neural process reliably affected by attention cues (Ora et al., 2015). Recorded by α scalp activity reflects a cortical state in which relatively little sensory information is received, which the gating mechanism of the thalamus may mediate. The decrease of power selectively occurs in tasks that need to pay attention to the processing of sensory information. Many studies support the view that high α-power reflects a reduction in (external) sensory processing (e.g., attention). Researchers found that the inefficiency or failure of the top-down function of the frontal lobe control area led to errors. They believed that errors might occur based on different neural mechanisms, such as a low level of continuous attention, a poor level of cognitive control, or an imbalance of the two levels (Shou et al., 2015). Before the wrong trail, the α-wave power of the temporomandibular area increased. In addition, O’Connell examined the temporal dynamics of cortical signals before continuous attention errors and found that the activity of the α-band began to increase about 20 s before the error occurred, indicating that the specific neural characteristics of top-down attention deficit were recorded by EEG 20 s before the error occurred (O’Connell et al., 2007).

In addition to ERP, more and more studies have found that some EEG band oscillations (such as the θ band) are related to error awareness. Some research found that ERN might partially manifest ongoing neural oscillations in the θ frequency band (Luu and Tucker, 2001). Further research proves that there is a strong alignment between θ band oscillation and ERN in time: that is, the phase of the θ band oscillation is reset to the same angle after each error, independent of the phase before the error, that is, the phase after the error θ frequency band oscillation is phase-locked (Luu et al., 2004). Previous studies using simulated EEG data have shown that in θ frequency band oscillation, ERN can be generated without phase locking (Yeung et al., 2010). According to the study, θ frequency band oscillation can be used as an effective indicator of error awareness (Navarro-Cebrian et al., 2013; Munneke et al., 2015; Murphy et al., 2015); that is, the perceived errors are more than those not the θ frequency band oscillates more.

ERN, Pe, and frequency band oscillation are important electrophysiological indicators for studying ground misconception. However, human error awareness’s physiological characteristics and mechanisms when performing tasks in high-altitude environments have not been studied. In order to explore whether the extreme flight environment will affect the EEG characteristics of pilots’ misconceptions, we recruited volunteers to collect brain electrophysiological signals on the ground through the improved double-choice Oddball paradigm. At the same time, we collected the EEG signals of pilots’ error awareness when performing task paradigms on the ground and in the flight environment. We analyzed EEG indicators sensitive to error awareness. We analyzed the EEG indicators sensitive to error awareness to determine the differences in EEG characteristics between pilots on the ground and the flight environment when they were in error awareness. This study provides theoretical guidance for the follow-up research on the intervention measures against error awareness and determines the target point positioning.

## Materials and methods

### Participants

A total of nine pilots who recently participated in the missions at the flight environment participated in the study (7 males, 2 females, age: 48.7 ± 6.3 years, age range: 41–57 years). In addition, we recruited 78 healthy volunteers (39 males, 39 females, age 40.3 ± 10.1 years, age range: 27–57 years) to participate in the ground-based experiment. All participants had normal vision or corrected to normal, non-color blindness, no severe astigmatism, was right-handed, self-reported, and had no history of mental and neurological diseases. Regarding education level, both pilots and volunteers have bachelor’s degrees or above. In addition, the socio-economic status of pilots and volunteers is middle class, with no requirements on professional background, work experience, etc. All the participants filled in the informed consent form before the experiment. These experiments were approved by the local ethics committee of the School of Biological Science and Medical Engineering at Beihang University. Participants were required to get enough sleep the night before the experiment. After the experiment, the participants received corresponding monetary rewards. Nine pilots conducted experiments on the ground and in the flight environment every 3 days, and each pilot repeated the experiment three times. On the other hand, 78 volunteers did only one experiment on the ground. Therefore, all 9 pilots and 78 volunteers were included in the EEG data analysis.

### Experiment task and process

**FIGURE 1:**
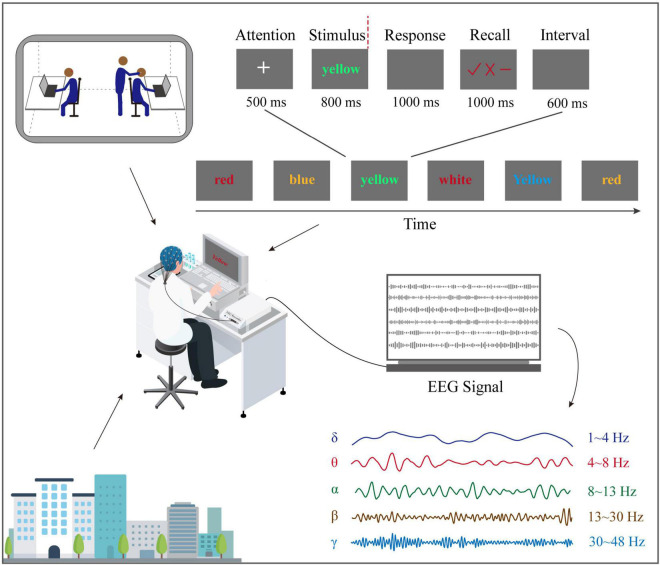
Experiment and task design. When volunteers and pilots perform double-choice oddball tasks on the ground or flight environment, EEG was recorded at the same time.

This study used a modified version of the error awareness task (EAT), essentially a double-choice Oddball task (Yuan et al., 2008). Each stimulus in the double choice Oddball task was centered on a black background in Times New Roman 72 font, with an angle of view of 1.6°, a brightness of 60 cd/m^2^, and a contrast of 80%. The stimulus is six kinds of color words (green, red, yellow, blue, purple, and white), as shown in Figure 1. Participants were asked to use the middle finger of their left hand to press the “D” key on the keyboard as quickly respond to each stimulus when the font color of the word was inconsistent with its meaning. Moreover, when the following two situations occur, use the left index finger to press the “F” key on the keyboard to respond. The first is that a word with the same meaning appears in two consecutive attempts, and the second is that its meaning is consistent with its color. After each stimulus response, three symbols (“√,” “×,” and “−”) will appear on the screen. At this time, the participants were required to make a subjective evaluation of the accuracy of the response just made (error awareness evaluation). The response is correct, error, and uncertain. The keys on the right hand correspond to the “L” key (“√”), “J” key (“×”), and “K” key (“−”).

Each test starts with a fixed white “+” and lasts 500 ms. After the “+” disappears, color word stimulation appears immediately and lasts 800 ms. Then the participants reacted to the stimulus, the stimulus interface disappeared, and a 1,000 ms black screen appeared. Next, the state of consciousness evaluation interface was presented for 1,000 ms. After the participants judged the accuracy of the response, the interface disappeared, and a 600 ms black screen was displayed. Finally, start the next try.

Before the formal experiment, to ensure that participants are familiar with the operating rules and reduce the number of unresponsive (too late to react), participants must complete 50 trial exercises. In the exercise phase, if the participants do not complete the response or error awareness evaluation within the specified time, they will receive the prompt “response is too slow, please speed up.” In addition, participants will receive feedback on the operation results after completing all trial exercises. For example, if the number of non-reactions exceeds five times during the exercise, they will give feedback, “too many times of non-reactions, please press any key to continue the exercise.” On the contrary, they will give feedback, “At the end of the exercise phase, please be prepared, and then the formal experiment will begin.” After the practice phase, volunteers began to conduct formal experiments. Then, participants completed 10 blocks containing 180 trials in the formal experiment, and a 2-min rest time was set between each block. Overall, a complete test lasted about 30 min.

### EEG recording

When performing the double-choice Oddball task on the ground and in-flight experiment, we used 64 Ag/AgCl chloride electrodes embedded in the elastic Lycra cap to measure continuous EEG activity in the cortex. However, only 10 PFC and 8 OPC electrodes were used for tests to save consumables and the time cost of testing. Place grounding electrodes in the center of the forehead, and install TP9-10 electrodes on the left and right earlobes to re-reference the data offline to the digitally linked earlobe (Berger et al., 2019). The conductive paste (Greentek, Inc., CHN) was moderately injected into the electrode position on the EEG cap of the brain to ensure that these impedances remained below 20 KΩ. All EEG signals were amplified by 64-channel amplifier systems (BrainAmp, brain products, GER). The digitization frequency is 1,000 Hz, the band-pass filtering frequency is 0.01–80 Hz, and the notch filtering frequency is 50 Hz.

### Types of trials

In order to avoid the influence of uncertain trial consciousness on the unaware trial, we added the “uncertainty of whether it is correct.” We divided the error awareness evaluation into three levels, namely “correct response,” “uncertain response is correct or not,” and “error response,” to obtain the error awareness of each trial reaction. According to the type of reaction, all reaction attempts were divided into six categories: aware errors, uncertain errors, unaware errors, aware correct, uncertain correct, and unaware correct. Many researchers in related fields believe that in the study of error processing, it is necessary to stack at least six times to get a reliable waveform (Olvet and Hajcak, 2010). Due to the human response and task characteristics, this study’s correct trial is easy to realize, and only a few uncertain and unconscious corrections are produced. However, due to the low signal-to-noise ratio, reliable waveforms cannot be obtained after superposition and averaging. Therefore, this study excludes correct consciousness and only analyzes three types of experiments: aware errors, uncertain errors, and unaware errors.

### Signal preprocessing

The EEGLAB software running on Matlab (v.2019b, MathWorks, USA) was conducted to preprocess EEG signals. First, the EEG signal was re-referenced to the average value of bilateral mastoid (TP9, TP10). FIR filter is used for 0.1∼35 Hz (12 dB/oct) band-pass filtering to remove artifacts caused by high-frequency EMG and slow voltage drift. Based on the reaction time, the continuous EEG data is divided into a time window from −500 to 1,000 ms. The baseline correction was performed using the period from −200 to −100 ms before the reaction. The segmented signal was visually inspected to delete the segments with more artifacts, and then the spherical spline was used to interpolate the channels with bad or more noise. Then, independent component analysis (ICA) was performed with clean segmented data, and the ICA components were identified from the EEG signals of each participant. The morphology, time course, and spectral characteristics of the ICA scalp were visually measured to identify and remove the components containing blink/eye movement or other artifacts.

### Event-related spectral perturbation

The EEG epochs from −500 to 1,100 ms generated event-related spectral perturbation (ERSPs) from −400 to 1,000 ms. For this paper, ERSPs were analyzed from 0 to 1,000 ms (post-stimulus onset) with a non-overlapping baseline of −400 to −100 ms (pre-stimulus onset) using the EEGLAB toolbox running under Matlab 2019b. Time-frequency decomposition was performed using the Morlet waveform implemented in the EEGLAB function newtimef.m. For the time-frequency representation of EEG data, the wavelet transformation using the Morlet waveform as a mother wavelet was chosen, resulting in a frequency resolution of approximately 1 Hz from 1 to 50 Hz. Baseline correction was done by a gain model. Each time-frequency time point was divided by the average pre-stimulus baseline power from −400 to −100 ms relative to stimulus onset at the same frequency. Bootstrap significance levels after FDR correction were calculated in ERSP analysis.

### Event-related potential analysis

In this study, the average absolute value of the maximum amplitude in the 0∼40 ms time window on FZ, FCZ, and CZ was defined as the ERN of the reaction-locking ERP component. This study analyzed Pe with different time windows, with Pe defined as the average absolute value of the maximum amplitude every 100 ms between 200 and 600 ms after the onset of the response measured on the Cz, CPz, and Pz electrodes. Only those in this study with at least 6 available trials for each error type were included in the analysis. According to the average waveform, a certain period around the peak value of the ICA component was selected as the time window for calculating the average amplitude.

Based on the defined time-frequency region of interest (e.g., θ and α), we calculated the mean value of the time-frequency maximum oscillation amplitude of various error awareness in a specific time-frequency region of interest and analyzed the ERSP difference of time-frequency representation between different error awareness. In this case, θ event-related oscillations (θ-ERO) (4∼8 Hz, −50∼150 ms), θ/α event-related desynchronization (θ/α-ERD) (4∼11 Hz, 180∼540 ms), and α event-related synchronization (α-ERS) (8∼13 Hz, 550∼950 ms) were selected as the time-frequency region of interest.

### Power spectrum analysis

We selected the period within 500 ms before the start of stimulation, used the Welch method to calculate the absolute power spectrum of EEG before stimulation corresponding to a different state of error awareness, and used the non-overlapping Hamming window with a window length of 1 s. We were in δ (1∼4 Hz), θ (4∼8 Hz), α (8∼13 Hz), β (13∼30 Hz), and γ (30∼48 Hz) frequency bands to calculate the power spectral density. The power spectral density values are normalized using decibel (dB) conversion.

### Statistical analysis

In the ground experiment of volunteers, the average number of aware errors, unaware errors, and uncertain errors was 196, 17, and 48, respectively. The average number of aware errors, unaware errors, and uncertain errors in pilots’ ground experiments was 187, 15, and 46, respectively. The average number of aware errors, unaware errors, and uncertain errors in pilots’ flight experiments was 201, 21, and 54, respectively.

Before looking at the effects of the individual signatures, a three-way ANOVA containing “Age” (youth: under 40 vs. middle age: over 41), “Sex” (males vs. females), and “Type” (unaware errors vs. aware errors uncertain errors) was conducted in volunteer samples. First, a three-way ANOVA containing “Sex” (males vs. females), “Test” (ground vs. flight), and “Type” (unaware errors vs. aware errors uncertain errors) was conducted in pilot samples. In addition, since all pilots are over 40 years old, the middle age group of volunteers was selected to compare with pilots tested in the ground environment, and a three-way ANOVA containing “Sex” (males vs. females), “Participant” (volunteers vs. pilots), and “Type” (unaware errors vs. aware errors uncertain errors) was conducted. Then, depending on which main effects/interactions are significant, ANOVAs were further used for *post hoc* analyses. Conditioned on significant *p* < 0.05, we conducted *post hoc* analyses with correction for multiple comparisons based on Sidak’s procedure.

For the awareness-type characteristics of volunteers who did experiments on the ground, we counted the changes of ERN, Pe, and power spectrum through one-way repeated measurement ANOVA. Based on the defined time-frequency region of interest, the average maximum oscillation amplitudes in the specific time-frequency region of interest for the three awareness types were analyzed by the one-way repeated measures ANOVA analysis. In addition, the ANOVA factor in the “Test” (ground vs. flight) was used to find the changes in Pe, α-ERS, and α-band power spectral density to analyze the difference in error awareness between ground and flight. In these statistical analyses, if the corrected *p* is less than 0.05, there is a significant difference.

## Results

### In the experiment of ground volunteers, there were differences in Pe, time-frequency region of interest, and power spectrum between multiple error awareness

**FIGURE 2:**
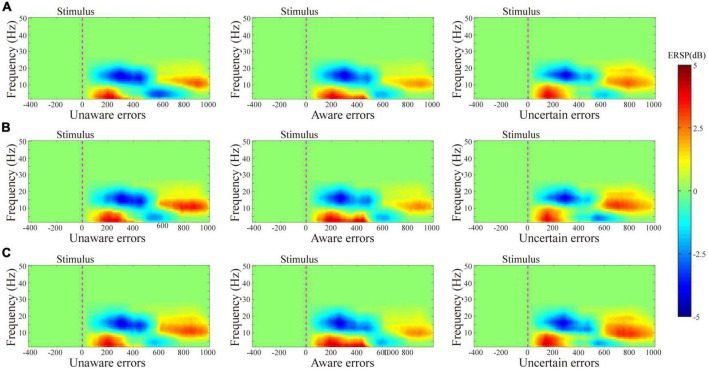
Results of the average ERSP. ERSP plot with masked unsignificant reactions (green area) shows values averaged for the whole group in each experiment. **(A–C)** Show the ERSP results from volunteers tested on the ground cabin, pilots tested on the ground cabin, and pilots tested in the flight environment, respectively.

**FIGURE 3:**
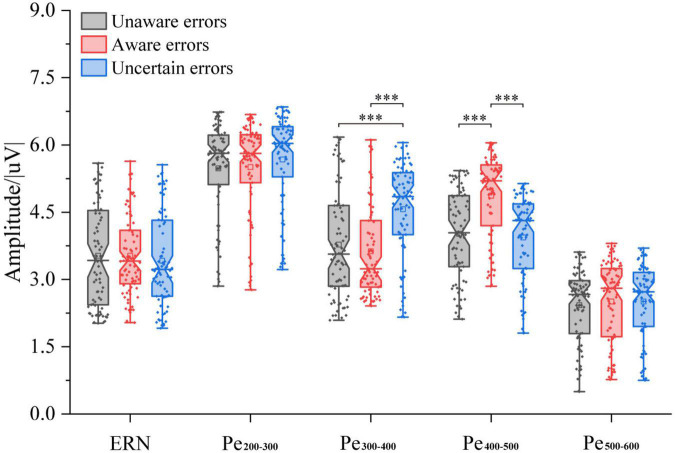
ERP amplitude of multiple error awareness in volunteers’ ground experiment (*n* = 78). The squares in the box plots indicated the mean values, the horizontal lines indicated the medians, and both ends indicated the maximum and minimum values (^***^p < 0.0001).

The results of the average ERSP are shown in Figure 2. The three-way ANOVA containing “Age,” “Sex,” and “Type” indicates that “Type” has a main effect in Pe_300–400_ (*F* = 16.10, *p* < 0.0001, η^2^ = 0.13) and in Pe_400–500_ (*F* = 19.80, *p* < 0.0001, η^2^ = 0.15), no interaction between these factors was significant. For ERN, the repeated measures ANOVA analysis of the three-awareness found no significant difference between the types of awareness. However, it can be seen from Figure 3 that there are differences in the amplitude of Pe between different consciousness in some time windows. For Pe, on the time window of 200–600 ms, the amplitude of uncertain error waveform in 300–400 ms is higher than that of unaware error (*F*_1/77_ = 21.75, *p* < 0.0001, η^2^ = 0.22) and aware error (*F*_1/77_ = 28.10, *p* < 0.0001, η^2^ = 0.27). In 400–500 ms, the amplitude of the aware error waveform is the largest, which is significantly different from the unaware error (*F*_1/77_ = 28.25, *p* < 0.0001, η^2^ = 0.27) and uncertain error (*F*_1/77_ = 41.37, *p* < 0.0001, η^2^ = 0.35).

**FIGURE 4:**
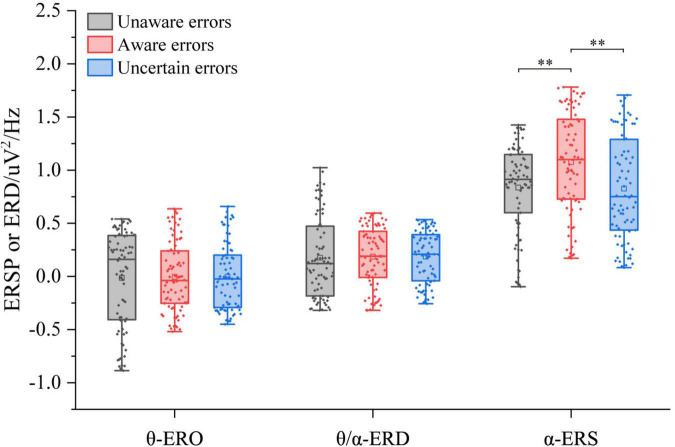
ERSP/ERD of multiple error awareness on the region of interest (*n* = 78). The squares in the box plots indicated the mean values, the horizontal lines indicated the medians, and both ends indicated the maximum and minimum values (^**^p < 0.001).

The three-way ANOVA containing “Age,” “Sex,” and “Type” indicates that “Age” and “Type” have main effects in α-ERS (Age: *F* = 6.40, *p* = 0.01214, η^2^ = 0.03; Type: *F* = 4.94, *p* = 0.0080, η^2^ = 0.04), an interaction between “Age” and “Type” (Age × Type: *F* = 5.81, *p* = 0.0035, η^2^ = 0.05) was significant. In the central frontal area, there were no significant differences in θ-ERO and θ/α-ERD of different error awareness responses (*p* < 0.05, Figure 4). About α-ERS, there were significant differences between different types of error awareness responses. Aware error was significantly higher than that unaware error (*F*_1/77_ = 11.95, *p* < 0.001, η^2^ = 0.13) and uncertain errors (*F*_1/77_ = 11.88, *p* = 0.0009, η^2^ = 0.13). There was no significant difference between uncertain errors and unaware errors.

**FIGURE 5:**
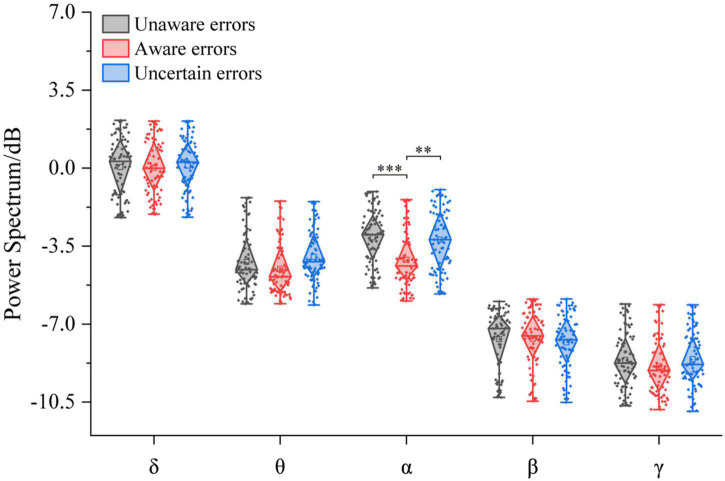
Power spectral density corresponds to multiple error awareness in each frequency band (*n* = 78). The squares in the box plots indicated the mean values, the horizontal lines indicated the medians, and both ends indicated the maximum and minimum values (^**^p < 0.001, ^***^p < 0.0001).

The three-way ANOVA containing “Age,” “Sex,” and “Type” indicates that “Age” has a main effect in δ power (*F* = 5.73, *p* = 0.0175, η^2^ = 0.03), and “Type” has a main effect in α power (*F* = 17.30, *p* < 0.0001, η^2^ = 0.14) and in γ power (*F* = 6.07, *p* = 0.0027, η^2^ = 0.05); no interaction between these factors was significant. The repeated measurement variance analysis results of the power spectral density of each frequency band are shown in Figure 5. In the α-band, the power spectrum of aware error was the largest, significantly different from unaware error (*F*_1/77_ = 23.11, *p* < 0.0001, η^2^ = 0.23) and uncertain error (*F*_1/77_ = 16.20, *p* < 0.001, η^2^ = 0.17). At the same time, there was no difference between unaware error and uncertain error (*p* > 0.05). There was no significant difference between error consciousness in both δ, θ, β, and γ bands (*p* > 0.05).

### There was no difference in EEG characteristics between pilots and volunteers on ground experiments, but pilots’ characteristics were more prominent in flight

**FIGURE 6:**
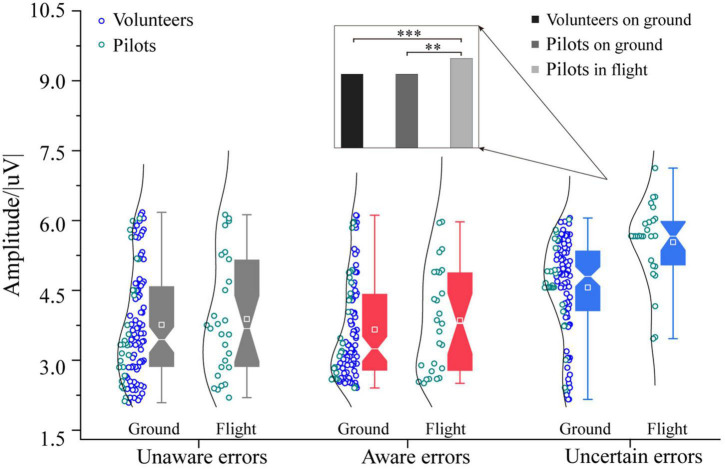
Differences in Pe (300–400) between pilots and volunteers in multiple error awareness on the ground or in flight environment. The squares in the box plots indicated the mean values, the white horizontal lines indicated the medians, and both ends indicated the maximum and minimum values (^**^p < 0.001, ^***^p < 0.0001).

The three-way ANOVA containing “Sex,” “Participant,” and “Type” indicates that “Type” has the main effect in Pe_300–400_ (*F* = 7.31, *p* = 0.0009, η^2^ = 0.08), and no interaction between these factors was significant. The three-way ANOVA containing “Sex,” “Test,” and “Type” indicates that “Sex,” “Test,” and “Type” have main effects in Pe_300–400_ (Sex: *F* = 6.20, *p* = 0.0139, η^2^ = 0.04; Test: *F* = 4.91, *p* = 0.0282, η^2^ = 0.03; Type: *F* = 13.32, *p* < 0.0001, η^2^ = 0.15), no interaction between these factors was significant. For the Pe amplitude on the 300∼400 ms time window, pilots in the flight environment have higher uncertain error consciousness than ground participants [*F*_1/(104, 26)_ = 20.49, *p* < 0.0001, η^2^ = 0.14, Figure 6]. The participants’ unaware and aware errors on the ground are no different from the pilots’ in the flight environment. There is no difference between the ground and the flight environment between unaware and aware errors. In order to further clarify the reasons for the difference in Pe_300–400_ between ground and flight, the individual differences between volunteers and pilots were excluded, and the uncertain error test results of volunteers and pilots on the ground were compared separately. The experimental results showed that the uncertain errors of volunteers in the ground experiment were no different from those of pilots (*p* > 0.05). In contrast, the uncertain errors of volunteers and pilots on the ground significantly differed from that of pilots in flight (Pilots in flight vs. Volunteers on ground: [*F*_1/(77, 26)_ = 18.23, *p* < 0.0001, η^2^ = 0.15]; Pilots in flight vs. Pilots on ground: [*F*_1/26_ = 14.43, *p* < 0.001, η^2^ = 0.36]. For the Pe amplitude on the 400∼500 ms time window.

**FIGURE 7:**
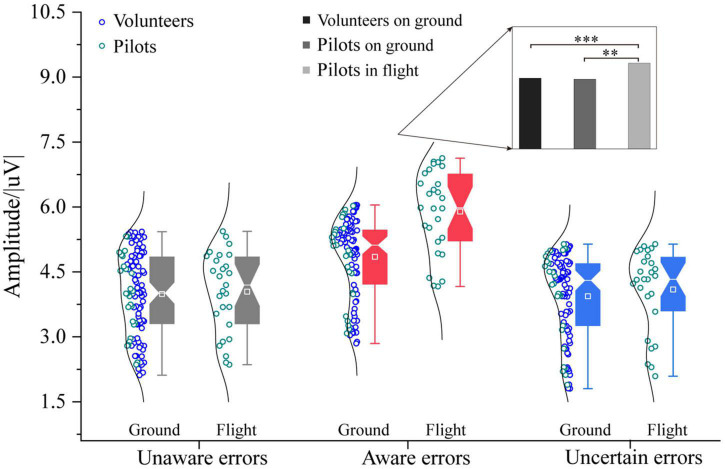
Differences in Pe (400–500) between pilots and volunteers in multiple error awareness on the ground or in flight environment. The squares in the box plots indicated the mean values, the white horizontal lines indicated the medians, and both ends indicated the maximum and minimum values (^**^p < 0.001, ^***^p < 0.0001).

The three-way ANOVA containing “Sex,” “Participant,” and “Type” indicates that “Type” has the main effect in Pe_400–500_ [*F* = 10.97, *p* < 0.0001, η^2^ = 0.12], and no interaction between these factors were significant. The three-way ANOVA containing “Sex,” “Test,” and “Type” indicates that “Test” and “Type” have main effects in Pe_400–500_ [Test: *F* = 10.75, *p* = 0.0013, η^2^ = 0.07; Type: *F* = 24.83, *p* < 0.0001, η^2^ = 0.25], an interaction between “Test” and “Type” (Test × Type: F = 3.86, *p* = 0.0233, η^2^ = 0.05) was significant. For the Pe amplitude on the 400∼500 ms time window, pilots in the flight environment have higher awareness errors consciousness than ground participants [*F*_1/(104, 26)_ = 27.05, *p* < 0.0001, η^2^ = 0.17, Figure 7]. Also, the participants’ unaware and uncertain errors on the ground are no different from the pilots’ in the flight environment. Further analysis found that the awareness errors of volunteers in the ground experiment were no different from those of pilots (*p* > 0.05). In contrast, the magnitude of the aware errors Pe_400–500_ by flight pilots was higher than that of ground volunteers and pilots [Pilots in flight vs. Volunteers on ground: *F*_1/(77, 26)_ = 23.91, *p* < 0.0001, η^2^ = 0.19; Pilots in flight vs. Pilots on ground: *F*_1/26_ = 20.00, *p* < 0.001, η^2^ = 0.44].

**FIGURE 8:**
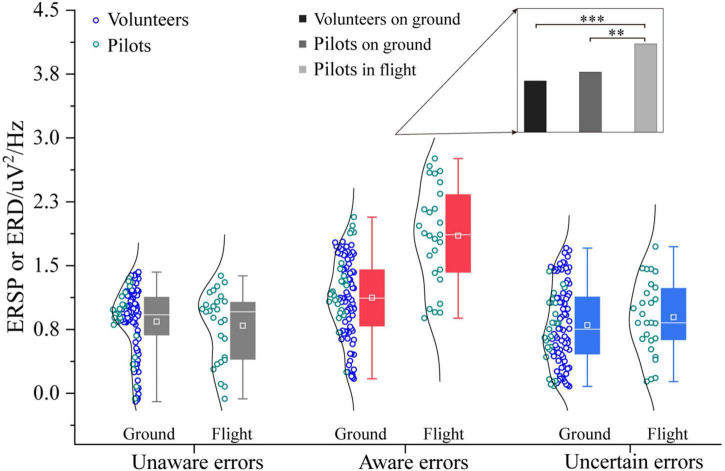
Differences in α-ERS between pilots and volunteers in multiple error awareness on the ground or in flight environment. The squares in the box plots indicated the mean values, the white horizontal lines indicated the medians, and both ends indicated the maximum and minimum values (^**^p < 0.001, ^***^p < 0.0001).

The three-way ANOVA containing “Sex,” “Participant,” and “Type” indicates that “Type” has the main effect in α-ERS (*F* = 5.04, *p* = 0.0075, η^2^ = 0.06), and an interaction between “Participant” and “Type” was significant (Participant × Type: *F* = 3.77, *p* = 0.0251, η^2^ = 0.04). The three-way ANOVA containing “Sex,” “Test,” and “Type” indicates that “Sex,” “Test,” and “Type” have main effects in α-ERS (Sex: *F* = 6.46, *p* = 0.0121, η^2^ = 0.04; Test: *F* = 5.63, *p* = 0.0190, η^2^ = 0.04; Type: F = 32.13, *p* < 0.0001, η^2^ = 0.30), an interaction between “Test” and “Type” (Test × Type: *F* = 5.47, *p* = 0.0051, η^2^ = 0.07) was significant. In the time-frequency analysis of volunteers’ ground experiments, it was found that the modulation related to error awareness mainly occurred in the frontal, central region. This chapter compares the average value of α-ERS maximum oscillation amplitude of pilots’ error awareness in flight with ground participants. The results showed that the average maximum amplitude of aware errors by pilots in the flight environment was higher than that of ground participants [*F*_1/(104, 26)_ = 47.38, *p* < 0.0001, η^2^ = 0.27, Figure 8]. However, there is no difference between the flight environment and the ground in the average value of the α-ERS maximum oscillation amplitude of the unaware and uncertain errors (*p* > 0.05). A depth classification analysis found that the average value of the maximum oscillation amplitude of α-ERS of pilots’ aware errors in the flight environment was significantly higher than that of ground volunteers and pilots (Pilots in flight vs. Volunteers on ground: *F*_1/(77, 26)_ = 47.66, *p* < 0.0001, η^2^ = 0.32; Pilots in flight vs. Pilots on ground: *F*_1/26_ = 20.24, *p* < 0.001, η^2^ = 0.44).

**FIGURE 9:**
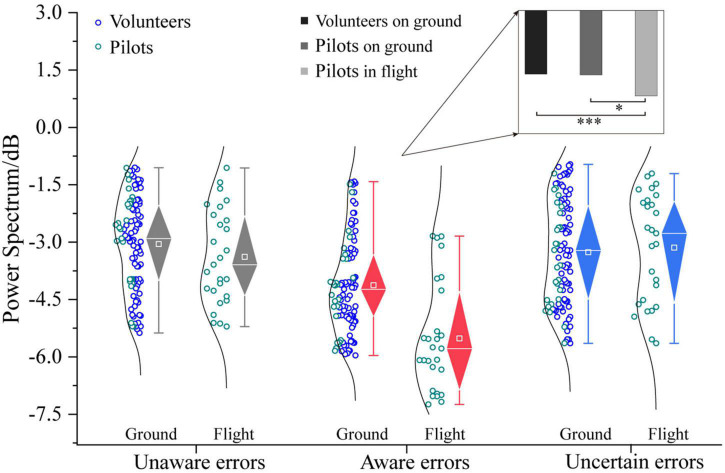
Differences in α-frequency band between pilots and volunteers in multiple error awareness on the ground or in flight environment. The squares in the box plots indicated the mean values, the white horizontal lines indicated the medians, and both ends indicated the maximum and minimum values (*p < 0.05, ^***^p < 0.0001).

The three-way ANOVA containing “Sex,” “Participant,” and “Type” indicates that “Type” has a main effect in α power (*F* = 16.11, *p* < 0.0001, η^2^ = 0.17), and no interaction between these factors was significant. The three-way ANOVA containing “Sex,” “Test,” and “Type” indicates that “Test” and “Type” have main effects in α power (Test: *F* = 6.07, *p* = 0.0149, η^2^ = 0.04; Type: *F* = 22.00, *p* < 0.0001, η^2^ = 0.23), an interaction between “Test” and “Type” (Test × Type: *F* = 3.43, *p* = 0.0349, η^2^ = 0.04) was significant. In the frequency band analysis of multiple error awareness in volunteers’ ground experiments, only the aware errors between the α-band differed from the unaware and uncertain errors. In order to further study the different effects of ground and flight environments on error awareness, this section analyzes the α-band power spectral density of error awareness of pilots in flight environments and participants on the ground. Figure 9 shows that the power spectral density of the α-band that pilots’ aware error of on the flight environment was higher than that of ground participants [*F*_1/(104, 26)_ = 24.77, *p* < 0.0001, η^2^ = 0.16]. However, there is no difference between the flight environment and the ground in the α-band power spectral density of unaware and uncertain errors (*p* > 0.05). Flight environment pilots realized that the aware error α-band power spectral density was the highest, which was significantly higher than that of ground volunteers and pilots [Pilots in flight vs. Volunteers on ground: *F*_1/(77, 26)_ = 22.27, *p* < 0.0001, η^2^ = 0.18; Pilots in flight vs. Pilots on ground: *F*_1/26_ = 13.71, *p* < 0.05, η^2^ = 0.35].

## Discussion

The task paradigm of this study, the dual choice Oddball task, improves the evaluation of only high-frequency stimulus responses in the standard EAT task, requiring participants to respond accurately and quickly to high-frequency standard and low-frequency discriminative stimuli, and different types of stimuli correspond to different responses. In addition, participants must suppress the dominant response to standard stimuli to ensure the correct response to discriminative stimuli. This method effectively controls behavior inhibition (Kong et al., 2015). At the same time, we divide the error consciousness from the traditional two categories into three categories to make the classification results more accurate. First, we separated “uncertain errors.” On the one hand, we can prevent low self-confidence errors from interfering with unaware errors. On the other hand, we can obtain the error awareness between aware and unaware errors, which is more conducive to studying the neural mechanism of error awareness. At the same time, in order to avoid the disadvantage of actively reporting errors, participants are more inclined not to report errors. In this study, the error reporting stage was modified to conduct the subjective evaluation of error consciousness after each trial response. Then, through the consistency of objective response accuracy and subjective evaluation, we can accurately obtain the error consciousness state of each response.

For ERN, the influence of various types of error awareness on ERN is not significant, which is consistent with the results of many previous studies on ERN, confirming that the modulation of error monitoring has nothing to do with error awareness (Nieuwenhuis et al., 2001; O’Connell et al., 2007; Endrass et al., 2010). For Pe, repeated measurement results show that the type of error awareness has a significant impact on Pe (Veen and Carter, 2002; O’Connell et al., 2007). This difference was reflected in all time windows within 300∼500 ms after the response, suggesting that Pe is associated with the states of error awareness. Among them, the amplitude of uncertain error response is the highest between 300 and 400 ms, and the amplitude of aware error response is the highest between 400 and 500 ms. Pe amplitude seems to indicate the participants’ feelings of participants, rather than the correctness of objective responses (Wang et al., 2020).

α wave is considered a sign of random changes in human attention. Many studies have reported that enhanced α-oscillation activity will affect visual and cognitive performance. The α-power of the parietal and occipital region can inhibit visual perception and identify the visual pathway of visual information. In the fast continuous visual presentation task, the α-power of the parietal, occipital region is negatively correlated with subjective attention level. In the attention orientation paradigm, the α-power of the parietal, occipital region is negatively correlated with visual spatial attention. Many EEG studies have shown that the enhanced power is a reliable signal of errors in various tasks. For example, in Go/Nogo tasks, the increased power before the Nogo trial can predict the failure of response inhibition (Mazaheri et al., 2010). These studies have shown that increased α-power is related to decreased attention. In addition, the alpha frequency band has also been implicated in the suppression and selection of attention (Hanslmayra et al., 2011; Klimesch, 2012). Previous studies have observed suppression of alpha activity after errors (Carp and Compton, 2009) and conflicts (Compton et al., 2011), suggesting higher alertness after these trials. These studies are all supported by the current research results; behavioral adjustment after realizing the mistake to prevent the mistake again and increased attention are manifestations of behavioral adjustment after the mistake.

The study found that the EEG characteristics of pilots’ error awareness in flight are more prominent than on the ground. First, most of the tasks performed in the flight environment have insufficient personnel and a heavy workload. The pilots’ spirit has been in a state of tension and anxiety for a long time, and they are worried about errors during the execution of the mission. As a result, the amplitude of Pe on the 300∼400 ms and 400∼500 ms time windows is much higher than on the ground when pilots are uncertain and aware of errors, respectively. At the same time, time-frequency analysis found that the modulation related to error awareness mainly occurred in the frontal and central regions. The amplitude of α-ERS oscillation was higher when the pilots of the flight environment realized the error. In the α-band, pilots are more aware of the wrong brain power spectrum. By identifying EEG characteristics when people are aware of errors when performing tasks, we can clarify the neural correlates of error awareness. Pilot status has been closely related to stress (Venus, 2020), common mental disorders (Feijó et al., 2012), emotional impairment (O’Hagan et al., 2020), depressive symptoms (Aljurf et al., 2018), and burnout (Demerouti et al., 2019). The research we know measures loneliness and cognitive ability, and the results show that loneliness is associated with an increased risk of cognitive decline (John and William, 2008). Early research found that compared with the control group, the subjects with higher scores in anxiety and worry had increased brain activity related to errors (Hajcak et al., 2003). Induction of transient stress in healthy adults is associated with increased blood flow in ACC (Kimbrell et al., 1999). Similarly, these results are consistent with imaging studies. Veterans with post-traumatic stress disorder have abnormal ACC activity during emotional Stroop tasks. Interestingly, the brain region with dysfunction was in the rostral “emotion” region of ACC (Shin et al., 2001).

The relevant sensitivity characteristic value of pilots’ error awareness in-flight environment is higher than ground participants, and its mechanism has not been clarified. One possibility is that the complexity of the work content and time constraints have a more significant impact on the pilot’s human error. Secondly, pilots are in an isolated environment for a long time in the flight environment, and their mental pressure is high. As a result, they are prone to anxiety and loneliness, which affect the cognitive function of pilots and produce more obvious brain characteristics. The neural mechanism of more sensitive brain features of pilots’ error awareness in flight environments needs further study. Next, we will study the intervention measures against error awareness to avoid and reduce the human error of pilots in the flight environment.

## Data availability statement

The raw data supporting the conclusions of this article will be made available by the authors, without undue reservation.

## Ethics statement

The studies involving human participants were reviewed and approved by the Local Ethics Committee of the School of Biological Science and Medical Engineering at Beihang University. The patients/participants provided their written informed consent to participate in this study.

## Author contributions

PZ: conceptualization, methodology, investigation, data acquisition, validation, software, formal analysis, visualization, writing—original draft and review and editing, validation, and data curation. JY: investigation, conceptualization, resources, and writing—review and editing. ZL: conceptualization, methodology, software, resources, supervision, and project administration. QZ: conceptualization, methodology, writing—review and editing, coordination, supervision, validation, and funding acquisition. All authors contributed to the article and approved the submitted version.
